# A GPR-based framework for assessing corrosivity of concrete structures using frequency domain approach

**DOI:** 10.1016/j.heliyon.2025.e42641

**Published:** 2025-02-11

**Authors:** Nour Faris, Ahmed K. Khalil, Mohamed A.A. Abdelkareem, Sherif Abdelkhalek, Ali Fares, Tarek Zayed, Ghasan Alfalah

**Affiliations:** a Department of Building and Real Estate (BRE), Faculty of Construction and Environment (FCE), The Hong Kong Polytechnic University, Hung Hom, Kowloon, Hong Kong; b Mechanical Engineering Department, Faculty of Engineering, Helwan University, Helwan, Cairo, Egypt; c Department of Mechanical Engineering, The Hong Kong Polytechnic University, Hong Kong; d Department of Architecture and Building Sciences, King Saud University, Riyadh, Saudi Arabia

**Keywords:** Corrosion, Ground penetrating radar, Non-destructive testing, Reinforced concrete structure, Short-time fourier transform

## Abstract

Ground-penetrating radar (GPR) is a prominent non-destructive testing (NDT) method for corrosivity evaluation in concrete structures. Most GPR interpretation methods rely solely on the absolute values of rebar reflection intensity, making them vulnerable to misinterpretation of the effects of complex factors. This study introduces a more comprehensive GPR data interpretation method, encompassing analysis in time and time-frequency domains. The developed method constitutes efficient GPR data collection and pre-processing, deep learning rebar recognition, and frequency domain analysis using the Short-Time Fourier Transform (STFT). The center frequency of rebar responses was normalized and depth-corrected to standardize the analysis method. The GPR condition mapping thresholds were optimized and validated using ground truth conditions from hammer tapping and reinforcement exposure of reinforced concrete walls. The method demonstrated superior performance compared to the traditional amplitude-based approach in detecting and quantifying the extent of corrosion-induced deterioration, with an average accuracy of 0.80 for active corrosion and 0.84 for active-corrosion with corrosion-induced delamination.

## Highlights

• A GPR framework is introduced for corrosion detection in concrete structures.
• Time and frequency domain analysis are combined for improved GPR data interpretation.
• Deep learning is utilized for accurate rebar recognition in GPR data.
• Higher accuracy is achieved than traditional methods in detecting concrete corrosion.

## 1 Introduction

**Fig. 1:**
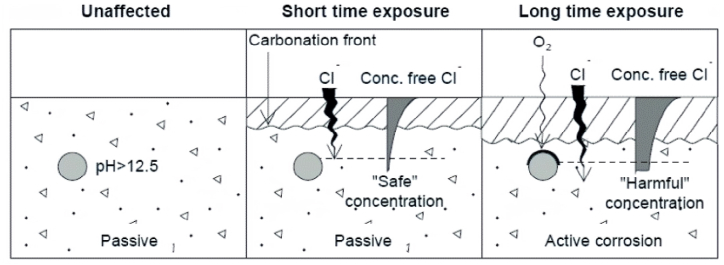
Corrosion initiation due to carbonation and chloride ions [4].

Reinforced concrete constitutes a crucial element of civil infrastructures [1]. It is imperative for the relevant authorities to uphold the concrete's state at levels that ensure both functionality and safety [2]. Corrosion is one of the most critical defects in concrete structures, as it directly affects their integrity and durability. The ongoing exposure of reinforced concrete components leads to an accelerated corrosion process of their reinforcement [3]. As illustrated in Fig. 1, the main contributing factor to corrosion is the exposure to moisture, chloride ions (particularly from sources like de-icing salts), and carbon dioxide [4,5]. The continuous exposure to rainwater and the condensation of warm, moist air on cooler surfaces can lead to moisture penetration. Insufficient insulation allows water droplets, along with chloride ions, to penetrate the concrete cover. This facilitates the electrochemical reactions that initiate and propagate corrosion on the steel. Progression of corrosion leads to the expansion of the rusted steel, generating internal tensile stresses that result in the cracking, deamination, and spalling of the neighboring concrete [6]. Indeed, corrosion compromises the structural integrity by undermining its ability to bear loads. Therefore, consistent examination and preservation are imperative in identifying and reducing the impact of corrosion.

Most inspectors assess the side effects of corrosion through visual observation of rust stains or by employing hammer-tapping tests to detect corrosion-induced delamination [7,8]. hammer-tapping test involves striking the concrete surface and analyzing the sound to identify delaminated areas. To improve the interpretation efficiency, Louhi Kasahara et al. [9] employed unsupervised learning to automate the hammering data interpretation. The Federal Highway Administration (FHWA) [10] reports that hammer sounding is particularly effective for identifying moderate to severe delamination, with a clear ringing sound indicating a sound deck and a dull or hollow sound indicating delamination. Thus, the hammer-tapping test is not efficient for detecting initial delamination in its early stages.

**Fig. 2:**
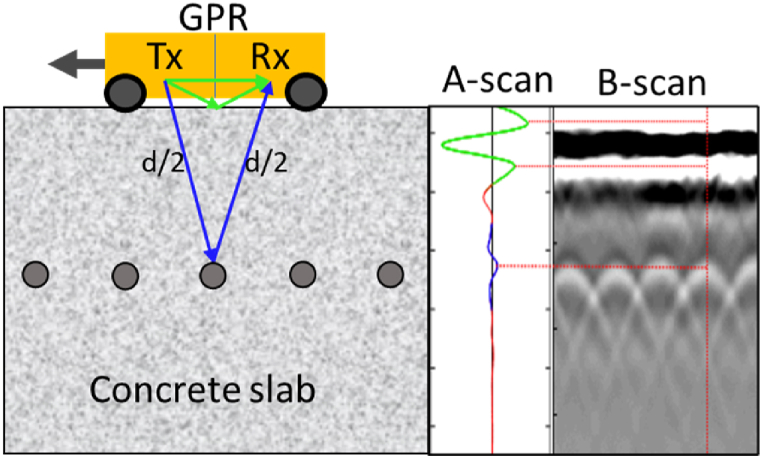
GPR basic principles.

Recently, ground penetrating radar (GPR) has emerged as a predominant non-destructive testing (NDT) for evaluating the corrosive nature of concrete structures [11]. The utilization of GPR became more prevalent among infrastructure operators and managers due to its capability to provide data swiftly and with a high level of spatial detail [12,13]. Furthermore, GPR can efficiently assess the condition of concrete elements in the presence of different overlay layers. Fig. 2 demonstrates the use of GPR electromagnetic waves (EW) in evaluating the internal structure of concrete, providing critical insights into potential corrosion within the reinforcement [11]. Since concrete is a nonmagnetic material, the concrete's magnetic permeability (μ) is assumed to be equal to the free space permeability (μ0) [14]. Thus, the electromagnetic wave attenuation (α), as described in Eq. (2), is primarily influenced by the electrical conductivity (σ) and the imaginary part of dielectric permittivity (${ℇ}^{\prime \prime }$) [12,13,15]. The corrosive environment primarily consists of conductive ions and moisture, which mainly impact the conductivity and permittivity of the concrete cover [12,13].

(1) $${ℇ}_{e}={ℇ}^{\prime }-i.{ℇ}^{\prime \prime }$$

(2) $$\alpha =\omega \sqrt{\;\frac{\mu_{0\;}ℇ}{2}}.\;\sqrt{\left(\sqrt{1+{\left(\frac{\sigma }{\omega ℇ}\right)}^{2}}-1\right)}\approx \frac{\sigma +{ℇ}^{\prime \prime }\omega }{2}+\sqrt{\;\frac{\mu_{0\;}}{{ℇ}^{\prime }\omega }}$$

Where, ${ℇ}_{e}$ is the effective dielectric constant, ${ℇ}^{\prime }\text{and}\;{ℇ}^{\prime \prime }$ are the real and imaginary parts of dielectric constant, *i* is the imaginary unit (*i* = $\sqrt{-1}$), σ is the electrical conductivity, μ_0_ is the magnetic permeability of free space, and ω is the angular wave frequency [16].

**Fig. 3:**
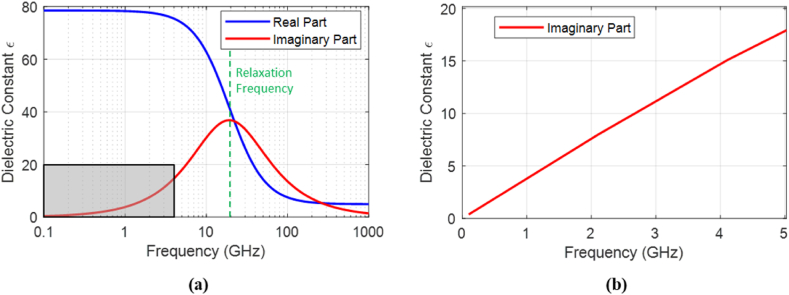
Debye function for real and imaginary parts of water dielectric constant.

In practice, the real and imaginary parts of the dielectric constant exhibit frequency-dependent behavior with significant variations near the relaxation frequency, as illustrated in Fig. 3 [17,18]. The relaxation frequency corresponds to the rate at which dipoles or charge carriers in a material can align with an alternating electric field [18]. Most materials do not have permanent dipoles and exhibit polarization due to electronic displacement, which occurs on short timescales, leading to very high relaxation frequencies of THz. On the other hand, polar materials such as water have molecules with permanent dipoles that need longer relaxation time to align with the applied electric field [19]. Thus, the relaxation frequency for water is significantly lower than nonpolar materials, at around 20 GHz. In the range of used GPR frequencies, concrete is considered a linear dispersive in the context of its dielectric properties due to its heterogeneous composition and the presence of water, aggregates, and cement paste. Accordingly, the concrete dielectric properties are frequency dependent, where the permittivity and conductivity change with frequency variation [20].

**Fig. 4:**
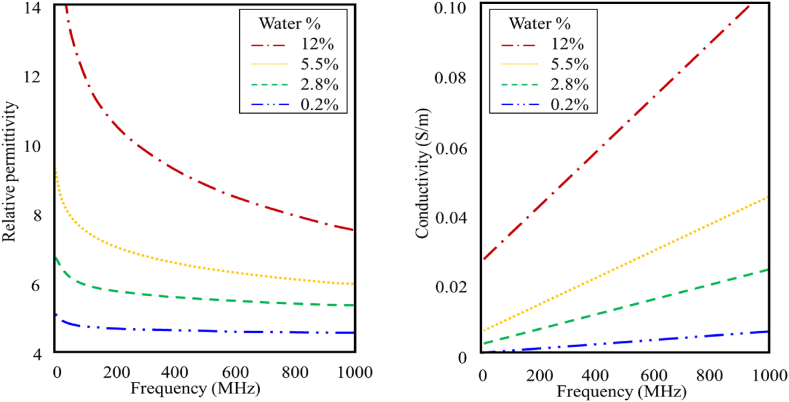
Conductivity and permittivity relationship with frequency and water content.

Frequency-dependent attenuation for the concrete increases in corrosive environments due to the elevated moisture content. Moreover, in the case of severely corroded concrete, corrosion byproducts cause interior cracks and delamination. The gap itself doesn't highly affect the GPR wave as the reflection from the air interface occurs almost at the same time as the reflection from the rebar due to the high speed of the electromagnetic wave in the air and the small thickness of the gaps, which is usually in millimeters [21]. However, these gaps increase the water capacity of the concrete cover and thus increase the frequency-dependent attenuation mechanism [22]. In addition, the polarization of water particles increases the conductivity of the charged ions in the water voids, and further increases the attenuation at high-frequency components of the wave, as illustrated in Fig. 4 [23]. The source waveform of some commercially available pulsed GPR systems is similar to a Ricker wavelet, which is a combination of a range of frequencies [20]. Accordingly, as the signal propagates in the corrosive environment, frequency-dependent attenuation causes the peak of the amplitude spectrum to shift to a lower frequency [18]. In this paper, the analysis and interpretation of GPR data in the context of corrosivity assessment investigate the shift in the peak of the amplitude spectrum from rebar reflection, hypothesizing that the corrosiveness of the concrete medium shifts the peak of the amplitude spectrum to a lower frequency.

## 2 Background

The analysis of GPR data for corrosivity assessment in the literature follows two primary approaches: the visual analysis of B-scan images and the numerical interpretation of A-scans. In visual interpretation, the analysis primarily relies on the expert's judgment to assess various patterns of reflections from rebars and categorize them into different conditions. The commonly observed B-scan patterns include distortions in the reinforcement layer, blurry areas, alterations or loss of slab boundaries, horizontal noise from delamination, vertical noise from cracks, and the visibility of the second rebar layer [[24], [25], [26], [27], [28], [29]]. Despite that visual interpretation of GPR data provides useful information to decision-makers, it heavily relies on expert's experience, which may introduce uncertainty and ambiguity in inspection outcomes. In addition, the impact of some factors can hardly recognized using this approach, such as rebar depth [27].

Conversely, numerical-based interpretation is more objective as it primarily relies on analyzing A-scans’ components. These components can be examined in either the time or time-frequency domains. Most researchers have focused on analyzing GPR data in the time domain due to its simplicity and intuitiveness [[30], [31], [32], [33], [34], [35], [36], [37], [38], [39], [40]]. Such analysis focuses on processing and clustering rebar reflections' amplitudes to map deteriorated areas. However, solely relying on the absolute values of the rebar reflection amplitude can lead to misinterpretations due to the complexity of the factors affecting the amplitude value [41]. These factors encompass the chloride [[42], [43], [44]]and moisture presence [29,45,46], rebar diameter and configuration [[47], [48], [49], [50]], surface and subsurface defects [29,45,46], corrosion byproducts [[51], [52], [53], [54]], and overlays [55,56]. The measured amplitude provides the resultant effect of these factors, which doesn't provide a full understanding of the causes of amplitude changes [57]. More recently, Hong et al. [58] used the values of amplitude, TWTT, and permittivity to provide more comprehensive corrosion detection using the numerical simulation of GPR. Moreover, P. T. wai Wong et al. [59] combined the amplitude analysis with the visual B-scan interpretation under the machine learning framework.

On the other hand, the frequency domain analysis involves investigating the changes in the amplitudes of different frequency components of the A-scans. Frequency components were usually investigated using S-transformation, which transforms the time domain into the time-frequency domain [[52], [53], [54],^60^,^61^]. Limited research has investigated the effect of corrosive environments on the frequency components of rebar reflections. For instance, Lai et al. [53] utilized the time-frequency domain analysis to characterize the reinforcement corrosion, hydration, and moisture content distribution in construction materials. The results indicated that moisture and chlorides reduce the peak frequency. Conversely, with the accelerated corrosion, the frequency slightly grew to a higher regime as the ions were forced to accumulate around the anode bar. Similarly, Lai et al. [54] used 1.5 and 2.6 GHz antennae to characterize the effect of the accelerated corrosion process on the A-scans in the time-frequency domain. The outcomes showed a sharp reduction in the center frequency of the rebar reflection after exposure to moisture and chlorides, followed by a slight increase during the accelerated corrosion process. However, the experimentation of Lai et al. [53] and Lai et al. [54] was limited to laboratory specimens and did not provide a quantitative assessment of concrete corrosivity.

In another study, Hong et al. [52] explored the effect of corrosive environment on the direct wave (DW) and reflected wave (RW) in time and time-frequency domains. The results indicated that the increased chloride and moisture content attenuated the DW and RW and shifted their frequency to the lower end. On the other hand, the accelerated corrosion increased the energy of rebar reflections. Accordingly, Hong et al. [52] suggested using the DW center frequency to map chloride content and RW energy to map corrosion. However, no quantitative methodology was suggested to evaluate the degree of corrosivity. In a similar vein, Hong et al. [61] combined amplitude and frequency attenuation mapping, proposing that regions with low center frequency and amplitude imply chloride contamination and potential active corrosion, while regions with high center frequency and normal amplitude suggest sound concrete. The investigation was also restricted to laboratory samples.

## 3 Problem statement and research objectives

The reviewed literature exhibits multiple attempts to evaluate the corrosivity of concrete using GPR. However, most of these studies relied on subjective visual interpretation or applied numerical data analysis in the time domain, relying solely on the peak amplitude values of rebar reflections. Only few studies have considered analysis in the time-frequency domain that takes the frequency-dependent behavior into consideration and provides more accurate information about the corrosivity of concrete cover [[52], [53], [54],^60^,^61^]. However, these studies were conducted on manufactured samples in laboratory environments, which may not accurately represent the natural corrosion process. Thus, the method's viability for practical industry applications remains to be explored. Furthermore, these studies have primarily focused on monitoring the behavior of EW without developing methodologies for quantitative corrosivity assessment. Additionally, there is a lack of a comprehensive time-frequency domain corrosivity assessment framework to facilitate the application of this method in extended concrete areas.

Based on the aforementioned discussion, this paper aims to bridge the existing gaps by presenting a practical method for quantitative evaluation of corrosion-induced deterioration using time-frequency domain GPR analysis in real-world scenarios. Accordingly, this paper developed an innovative method to automate and streamline the processes for rebar detection, data pre-processing, frequency-based analysis, and corrosivity mapping. The method employs deep learning (DL) to automatically detect rebar reflections in GPR data. Afterward, it incorporates novel GPR data pre-processing and filtering to prepare the data for the subsequent analysis. Subsequently, a standardized frequency domain analysis was adopted to achieve robust GPR condition mapping. The thresholds for GPR condition mapping were optimized by correlating GPR condition mapping with ground truth conditions obtained from acoustic delamination testing and visual evaluations of exposed reinforcement. The proposed framework addresses the limitations of existing GPR data processing and interpretation approaches, representing a significant advancement in non-destructive corrosivity evaluation.

## 4 Research methodology

**Fig. 5:**
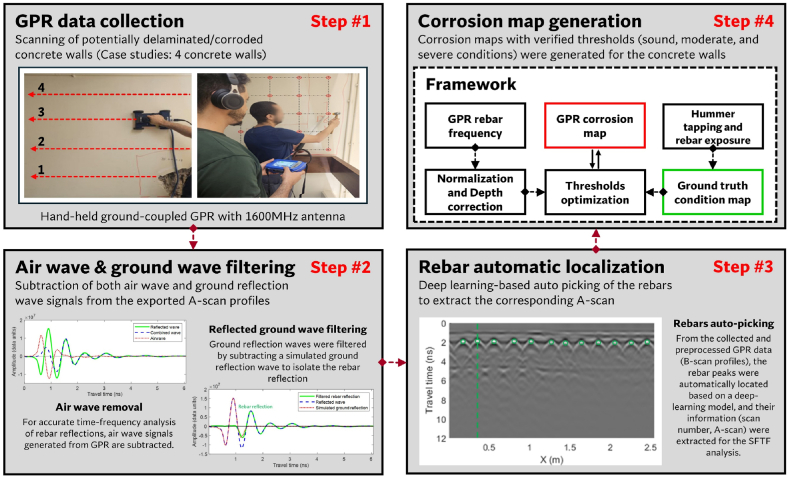
Research methodology.

This study developed a GPR-based method for quantifying corrosivity severity and extent in concrete structures using time and time-frequency domain analyses of GPR scans. As outlined in Fig. 5, The framework consists of four key stages: (1) data collection, (2) data pre-processing and filtering, (3) deep learning-based rebar localization, and (4) frequency domain analysis and corrosivity mapping.

The data preprocessing and filtering steps included band-pass filtering, DC offset removal, and filtration of air and ground waves. These preprocessing measures were applied to the raw GPR signals to enhance the reliability of the frequency-domain analysis. The frequency-domain analysis was standardized using normalization and depth correction, while the condition mapping thresholds were optimized based on ground truth delamination and corrosive conditions. Additionally, the DL-based rebar localization served as the backbone for automating the analysis framework. Comprehensive elaboration on each step of the framework is provided in the subsequent sections for clarity and in-depth understanding.

### 4.1 Data collection and pre-processing

**Fig. 6:**
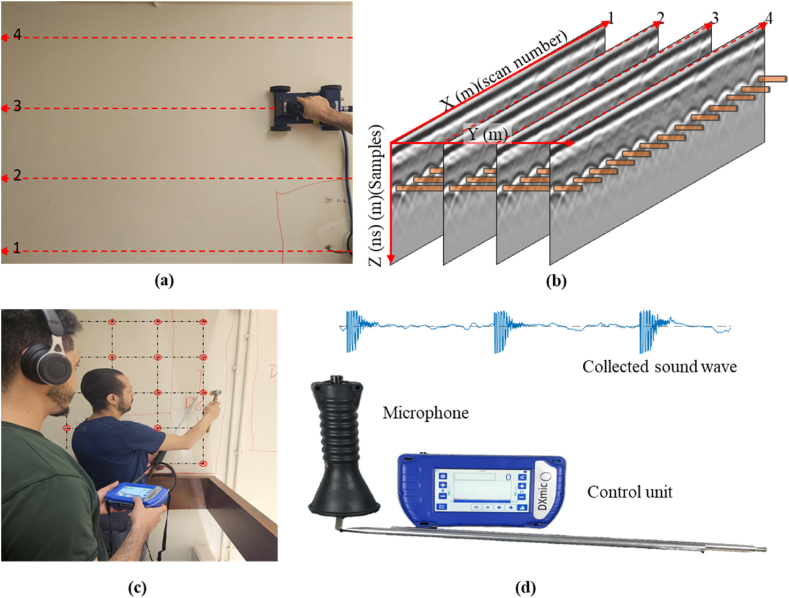
Data collection: (a) GPR scanning, (b) GPR data, (c) hummer sounding, and (d) sound data and microphone.

Four reinforced concrete walls, with clear concrete cover ranging between 3 and 4 cm and exhibiting delamination and corrosion, were chosen as case studies for this paper. During the data collection phase, a handheld ground-coupled GPR system equipped with a 1600 MHz antenna (GSSI 51600S) and operated through the GSSI SIR-4000 control unit was used to scan the reinforced concrete walls, as shown in Fig. 6 (a). Thereafter, the scans were exported using RADAN and processed in MATLAB, as depicted in Fig. 6 (b). The pre-processing mainly included time correction and band-pass filtering to eliminate the influence of extraneous signals. Moreover, the ground truth delamination and corrosion were evaluated through rebar exposure and hammer sounding, where the echo of the hammer tapping test was collected using a DXMIC PRO microphone to ensure precise analysis, as shown in Fig. 6 (c & d).

### 4.2 GPR waves filtering

Rebar reflection wavelets were examined in the time-frequency domain in order to study the changes in the center frequency. However, for shallow cover depths, the direct coupling and reflections from the surface may interfere with the rebar reflection and affect the wavelets’ frequency components. Assuming that the average speed of EW in the concrete medium is 10.5 cm/ns [62], using Eqs. (3), (4), the wavelength (*λ*) of the used 1.6 GHz GPR antenna is estimated to be 6.56 cm. Therefore, when the cover depth is less than 6.56 cm, the rebar reflections overlap with the direct coupling and reflections from the surface.

(3) $$\lambda =\frac{v}{f}$$

(4) $$v=\frac{c}{\sqrt{{ℇ}_{r}^{\prime }}}$$

Where ${ℇ}_{r}^{\prime }$ is the dielectric constant, mainly controlling the EW propagation velocity (*v*), and *c* is the EW velocity in free space (3 × 10^8^ m/s).

**Fig. 7:**
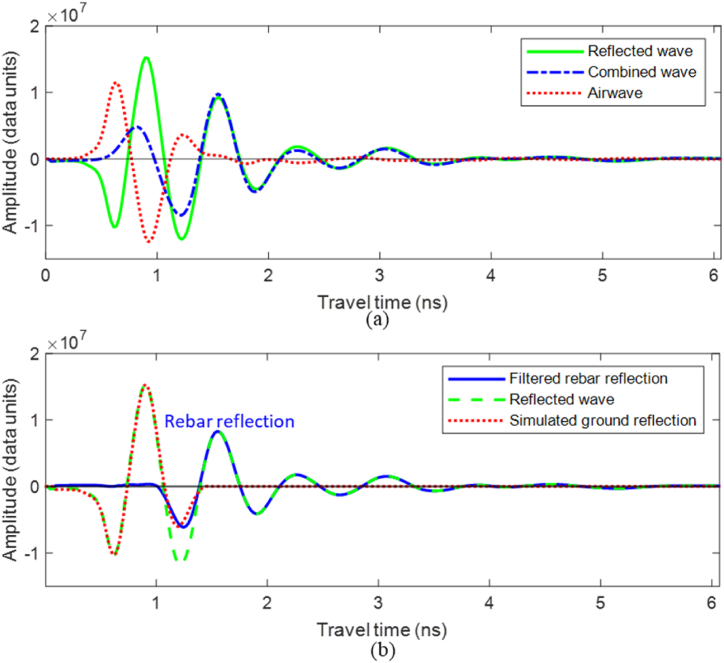
Wave filtering: (a) airwave removal and (b) ground wave removal using a simulation filter.

Accurate amplitude values at each TWTT step are essential for the time-frequency analysis of rebar reflections. Thus, when the GPR wavelength exceeds the concrete cover thickness, it is crucial to separate the rebar reflection from overlapping ground reflections to avoid compromising the accuracy of the frequency analysis. As depicted in Fig. 7, the ground-coupled GPR antenna concurrently receives two distinct reflections: one reflected from the concrete surface (reflected wave) and the other from the direct coupling between the transmitting and receiving antennas (airwave). Due to the small time difference between these waves, they merge into a combined wave. Thus, firstly, the airwave was subtracted from the A-scans, as illustrated in Fig. 7(a). The airwave was collected by directing the antenna to the sky, where no reflections from any objects exist. Subsequently, the ground reflection was filtered out by subtracting a simulated ground reflection wave to detach the rebar reflection, as illustrated in Fig. 6(b). The sequential removal of air and ground reflection ensures that the filtration adapts to changes in the coupling distance and surface properties.

The ground reflection from the concrete surface travels through the air gap between the antenna and the concrete surface. Air is considered non-magnetic and non-dispersive. Thus, the concrete surface reflections exhibit the characteristics of a linear time-invariant (LTI) system [63]. Accordingly, the concrete surface reflection Rc(t) can be simulated using Eq. (5). The reference incident x(t) was collected by capturing the reflection from a plain concrete surface and subtracting the direct air wave from the reflection. Subsequently, as illustrated in Eq. (6), the reflection coefficient R1 value was computed by dividing the amplitude of the first peak of the ground reflection by the amplitude of the first peak of the reference incident. Finally, the time position of the simulated ground reflection was set to match the location of its first peak with the first peak of the ground reflection.

(5) $$Rc\left(t\right)=x\left(t\right)\cdot R_{1}$$

(6) $$R_{1}=\frac{A_{\text{peak}1}}{A_{\text{reference}\;\text{wave}\;\text{peak}1}}$$

### 4.3 Autoamtic rebar localization

The rebar reflection coveys valuable information about the reinforcement and concrete cover condition. Rebar picking is one of the most time-consuming processes in GPR data analysis in the context of corrosivity evaluation. Thus, to make the method convenient for extensive applications, an automated framework encompassing automated DL rebar recognition was established in this paper, as depicted in the methodology in Fig. 5. This method involves the automatic localization of rebars within the B-scans via DL terminology. Subsequently, A-scans corresponding to rebar locations are designated for subsequent analysis using STFT.

**Fig. 8:**
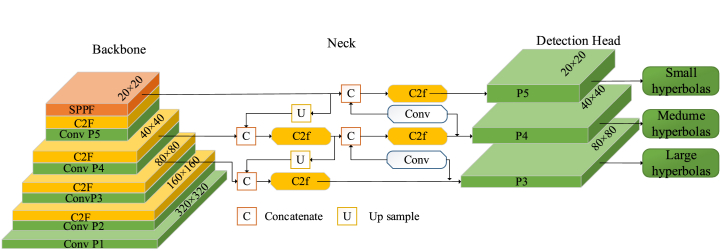
YOLOv8 architect.

In this study, YOLOv8 DL algorithm has been used to automate the rebar-picking task. YOLOv8 is the state of the art in object detection and classification. As illustrated in Fig. 8, the architect of this DL model mainly encompasses the neck, head, and backbone. Overall, the model constitutes 225 layers, 11138293 gradients, 11138309 parameters, and 28.7 GFLOPs [64]. The competence of YOLOv8 offers a promising DL network for rebar picking.

To develop the DL rebar picking model, 10125 rebar annotations were labeled to train and validate the model. A set of metrics was used to provide a thorough assessment of the model's performance in detecting rebar signatures. For example, Precision (P) represents the ratio of true positive (TP) predictions to the total predictions, as illustrated in Eq. (7). Recall (R), depicted in Eq. (8), represents the ratio of TP to the total relevant instances. Additionally, mean Average Precision at an IoU threshold of 0.5 (mAP50) assesses detection accuracy by averaging P across predictions, as described in Eq. (9). The Intersection over Union (IoU), shown in Eq. (10), compares the overlap between predicted and ground truth bounding boxes to their total area, measuring the model's ability to localize objects accurately.

(7) $$P=\frac{TP}{TP+FP}$$

(8) $$R=\frac{TP}{TP+FN}$$

(9) $$\text{mAP}50=\frac{1}{N}\sum_{i=1}^{N}{\text{AP}}_{i50}$$

(10) $$\text{IoU}=\frac{\text{Area}\;\text{of}\;\text{Overlap}}{\text{Area}\;\text{of}\;\text{Union}}$$

Where ${\text{AP}}_{i50}$ represents the average precision for the *i*_*th*_ class at 0.5 *IoU* threshold, and *N* denotes the total number of classes.

### 4.4 Time-frequency domain analysis

Frequency domain analysis provides detailed insights into the wave behavior in the concrete medium. This study investigates and quantifies the effect of the concrete medium's corrosivity on the frequency component of rebar-reflected wavelets. The Discrete Fourier Transform (DFT), typically computed using the Fast Fourier Transform (FFT), analyzes the overall time domain signals of the GPR scans, including reflections from various interfaces beyond the rebar. To focus on the frequency content of wavelets reflected from reinforcement, this study employed the Short-Time Fourier Transform (STFT), which is well-suited for non-stationary signals like GPR A-scans, where frequencies vary over time. STFT divides the A-scan into overlapping segments and applies the Fast Fourier Transform (FFT) on each segment. Discrete STFT, illustrated in Eq. (11), was used to analyze the discrete GPR A-scans.

(11) $$X\left[k,m\right]=\sum_{n=m}^{m+\left(N-1\right)}w\left[n-m\right]\cdot x\left[n\right]\cdot e^{-j2\pi k\left(n-m\right)/N}$$

Where *X*[*k,m*] is the STFT of the signal at time index m and frequency bin k, *x*[*n*] is the A-scan input signal, *w*[*n−m*] is a window function of length *N* starting from the sample at index *m*, *k* is the frequency bin index, $e^{-j2\pi k\left(n-m\right)/N}$ is a complex sinusoidal waveform with frequency *k* and phase indexed by *n* and *m* into the discrete-time and frequency domains.

**Fig. 9:**
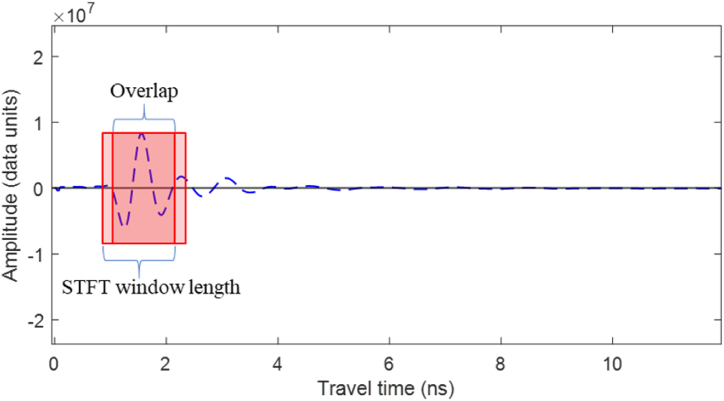
STFT window and overlap length.

To ensure capturing all frequency components in the rebar reflection, the STFT was applied at a window length equivalent to the size of the 1.6 GHz GPR antenna's mother wave. The number of samples in the window (N) is calculated using Eq. (12), where the sampling rate ($f_{s})$ is the number of samples per second, and $f_{c}$ is the GPR antenna nominal central frequency. As illustrated in Fig. 9, the overlap length was one sample shorter than the window length, so the window moves one sample each step to ensure the capturing of the entire rebar reflected wavelet.

(12) $$N=\left(\frac{1}{f_{c}}\right)\times f_{s}$$

### 4.5 Frequency mapping

#### 4.5.1 Normalization and depth correction

Following the STFT analysis, the frequency components of rebar reflection were examined. Center frequency values were collected at each rebar to monitor shifts from the nominal center frequency of the used GPR antenna and link them with the corrosivity of the concrete cover. To standardize the analysis, the rebar reflection frequency values ($f_{ci})$ were normalized based on the GPR antenna's nominal center frequency $\left(f_{cn}\right)$ in accordance with Eq. (13).

(13) $${Nf}_{i}={100∙\log}_{10}\left(\frac{f_{ci}}{f_{cn}}\right)$$

**Fig. 10:**
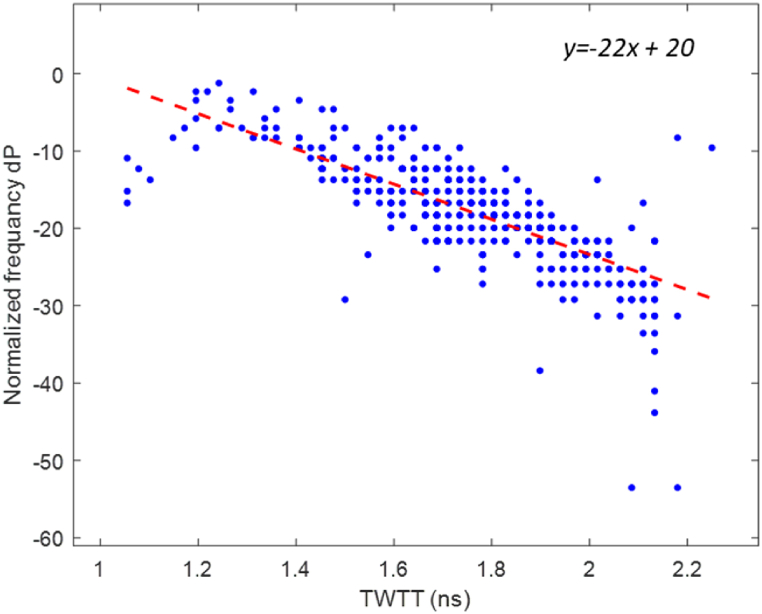
Reference regression between travel time and normalized frequency.

Cover depth variations affect the frequency behavior of the rebar reflections [65]. Thus, a correlation was formulated between the normalized frequency and travel time based on data collected from 4 different concrete walls, as illustrated in Fig. 10. Based on this regression, the variations in cover depth were addressed by calculating the equivalent frequency shift at 1 ns travel time using Eq. (14). This approach effectively mitigates the impact of cover depth variations, ensuring that the normalized frequencies are brought to a consistent scale for meaningful analysis.

(14) $${DCf}_{i}={Nf}_{i}-\left[\left({TWTT}_{i}-1\right)∙A+B\right]$$

Where $f_{ci}$ is the ith rebar's central frequency, $f_{cn}$ is the nominal center frequency of the used GPR antenna, ${Nf}_{i}$ is the normalized center frequency of ith rebar in dB, ${DCf}_{i}$ is the depth-corrected normalized center frequency of the ith rebar, ${TWTT}_{i}$ is the two-way travel time to the ith rebar, A is the slope of the regression line, and B is the regression intercept value.

#### 4.5.2 Threshold optimization

**Fig. 11:**
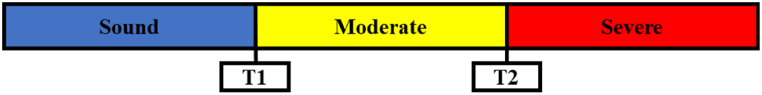
Normalized GPR center frequency thresholds for concrete conditions of Sound, Moderate (corrosive concrete), and Severe (corrosion-induced delamination).

Proper thresholding is essential to generate an accurate GPR condition map that enables precise identification of deteriorated areas within concrete structures. Self-supervised clustering techniques, such as K-means clustering, have proven to be unreliable because the thresholds for these methods vary significantly from one case to another. This can lead to either overestimation or underestimation of the condition, making them less effective for consistent and accurate clustering in various scenarios [41]. Thus, this study established standardized thresholds based on the goodness of the correlation between the GPR condition map and ground truth data from sound delamination test and rebar exposure. Two optimized threshold values were determined to categorize three conditions within the concrete structure. As depicted in Fig. 11, the first threshold separates corrosive areas from sound areas, while the second threshold distinguishes regions with severe corrosivity where corrosion-induced delamination is anticipated. To optimize and validate the GPR thresholds, the ground truth delamination and corrosivity map were generated using hammer sounding and rebar exposure. Accordingly, the GPR mapping thresholds were selected to achieve the best agreement with the ground truth condition.

## 5 Results and discussion

The methodology was applied to the data from the four concrete walls. Firstly, the thresholds were optimized using data from one concrete wall to achieve the best agreement between the GPR condition map and the ground truth data. Second, the optimized thresholds were tested on another three concrete walls to validate the accuracy of the developed method.

### 5.2 Thresholds optimization result

The method was applied on one of the concrete walls to optimize the GPR condition mapping thresholds.

### 5.1 Automatic rebar picking performance

**Fig. 12:**
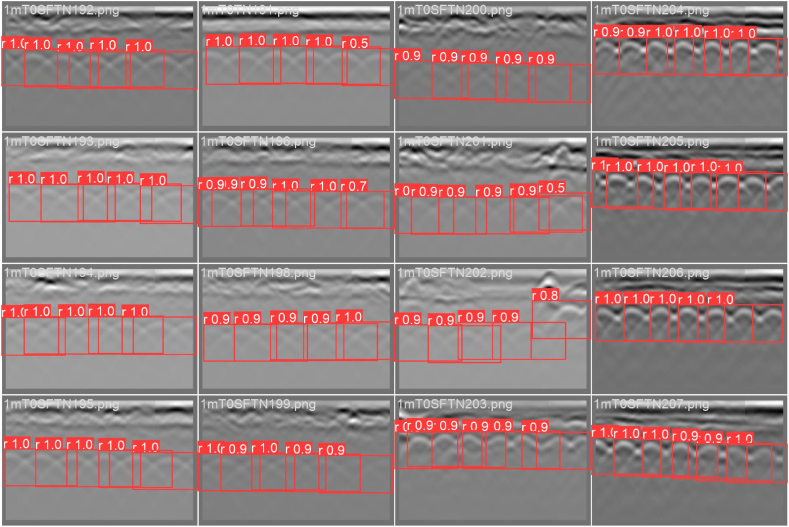
Sample of the validation patch.

The model was trained on a laptop with i7-12700H CPU and RTX 3060 6 GB GPU. The model was trained and validated on 10,125 rebar annotations divided manually into 80 % training and 20 % validation to prevent overfitting. As depicted in Fig. 12, the model was trained for 100 epochs with a 16-image batch size and 0.0005 decay weight.

**Fig. 13:**
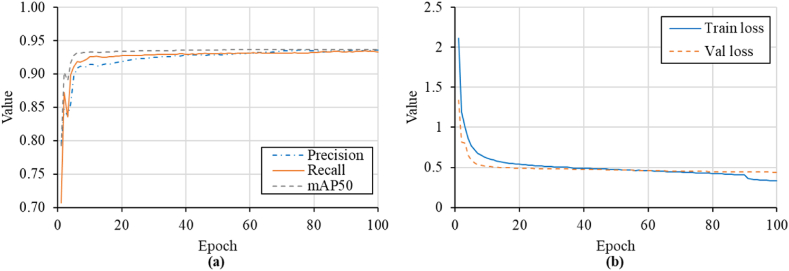
DL model performance: (a) precision, recall, and mAP50, and (b) training and validation losses.

The GPR training data was obtained from a concrete deck that encompasses highly deteriorated areas with blurry rebar signatures. Nonetheless, the model has achieved remarkable accuracy in recognizing the rebar signatures in the deteriorated areas. As illustrated in Fig. 13, the model exhibited a rapid decline in both training and validation losses during the first ten epochs, followed by a more gradual decrease throughout the remaining epochs. During the training and validation process, both precision and recall showed strong performance, improving from 0.80 to 0.71 to 0.94 and 0.93, respectively. Similarly, the mAP50 metric exhibited excellent progress, steadily increasing to 0.94. Finally, an interactive window was employed to add the unrecognized rebar signatures.

#### 5.2.1 GPR condition mapping

**Fig. 14:**
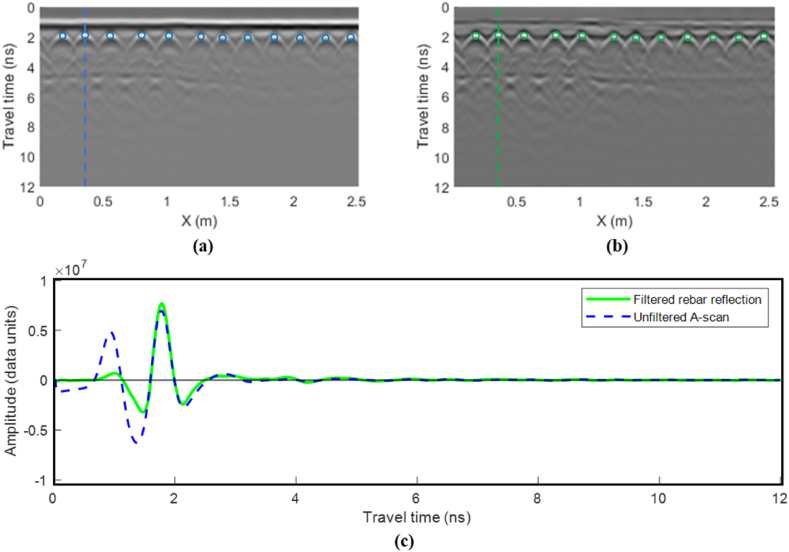
GPR data processing and rebar picking; (a) unprocessed B-scan, (b) filtered B-scan, and (c) A-scan sample at rebar location before and after filtering.

The GPR data was first subjected to band-pass filtering. Afterward, the scans were filtered by subtracting the airwave and the simulated ground reflection. Fig. 14 illustrates the performance of the filtering algorithm on a 2.5 m B-scan that includes 12 rebar reflections. As illustrated in Fig. 14 (c), the filtering algorithm effectively isolated the airwave and ground reflection, ensuring that the wavelets reflected from the rebars were accurately captured. This process guarantees precise time-frequency analysis. Consequently, the rebar locations were automatically identified to extract the A-scans at these locations to apply frequency analysis on the filtered rebar reflections, as illustrated in Fig. 14.

**Fig. 15:**
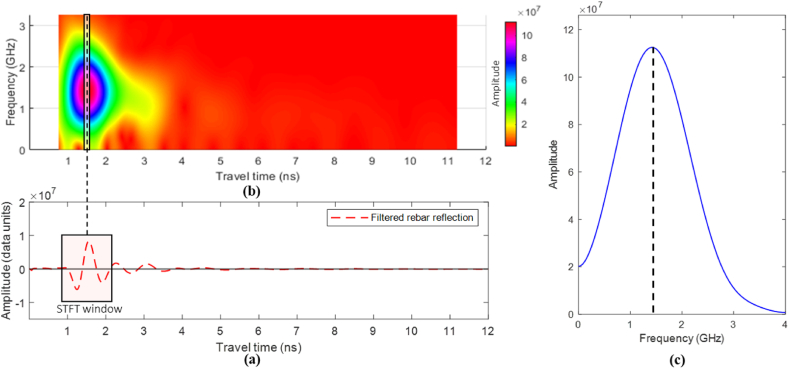
Time-frequency analysis on sample A-scan. Where (a) is a sample STFT window on a filtered rebar reflection, (b) is the time-frequency spectrogram, and (c) is a slice in the time-frequency spectrum at the rebar location.

The STFT analysis was applied to the filtered A-scans extracted at rebar locations. As shown in Fig. 15, the filtered rebar reflections were subjected to STFT with a window length equal to the mother-wave length (1.25 ns). The window moves one sample at each step. The STFT at each step in Fig. 15(a) corresponds to a vertical slice in the spectrum in Fig. 15(b). The vertical slice from the window encompassing the rebar reflection is depicted in Fig. 15(c).

This study hypothesizes that the corrosive environment attenuates the high-frequency components, resulting in lower frequencies dominating the reflected signal. Moreover, corrosion-induced cracks and delamination create gaps or voids that can trap more moisture and conductive ions that exacerbate the corrosion condition and further attenuate the high-frequency components of the waves. Indeed, the analysis results indicate that the center frequency of the rebar reflections systematically decreases in areas where active corrosion presents. This reduction in center frequency was considered a key indicator of deterioration, as it reflects changes in the material properties and structure of the concrete cover.

**Fig. 16:**
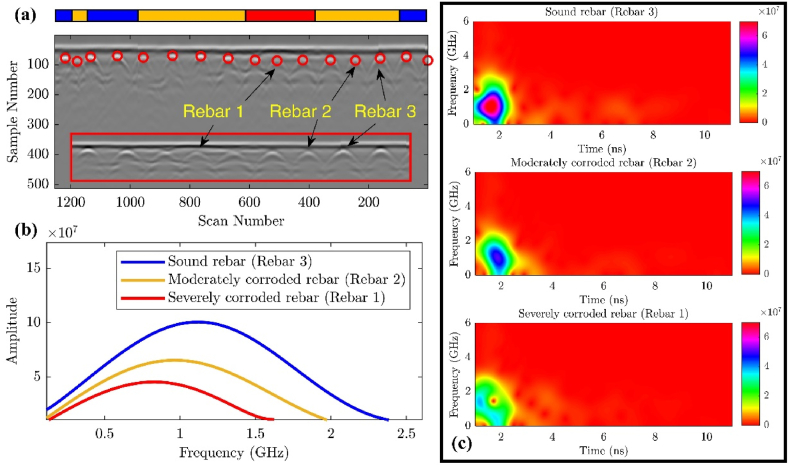
STFT analysis: (a) sample B-scan indicating rebar with different conditions; (b) Frequency components of rebars' wavelets; (c) time-frequency spectrum of the rebars.

In Fig. 16(a), the B-scan clearly shows that the reflections from the rebar located in delaminated and corroded regions are visibly blurred and distorted when compared to reflections from sound areas. A more detailed time-frequency domain analysis, as shown in Fig. 16(b) and (c), reveals that both the amplitude and center frequency of the rebar reflections are closely related to the degree of corrosivity. As the corrosivity increases, the amplitude of the reflected signal diminishes due to energy absorption by the corrosive materials. Simultaneously, the center frequency shifts toward lower values, which is a clear indication that the deterioration of the rebar and the surrounding concrete is affecting the propagation of higher-frequency components. This relationship between frequency shift and corrosivity provides a quantifiable measure for assessing the extent of deterioration within the structure.

**Fig. 17:**
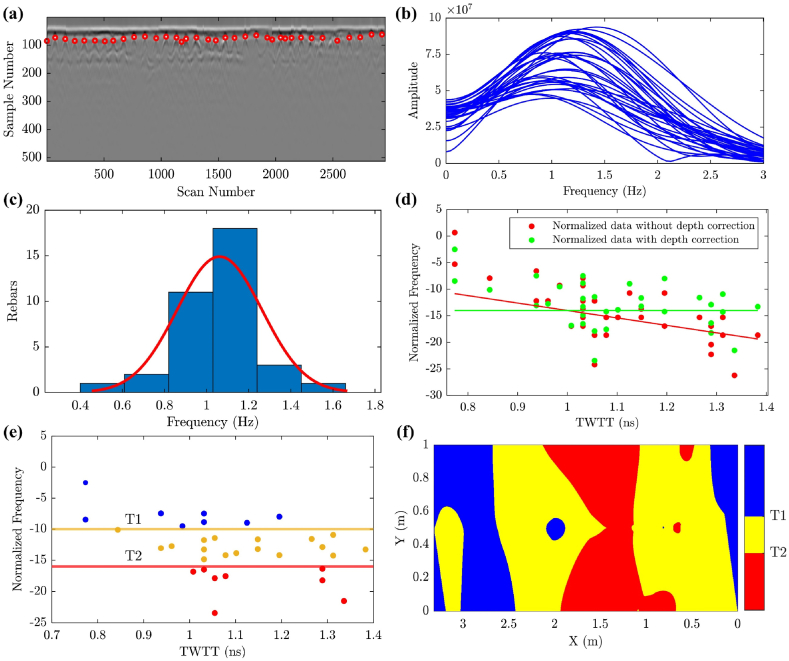
GPR frequency condition mapping steps: (a) Automated rebar detection; (b) Frequency domain representation of rebar wavelets; (c) Histogram presenting the Probability Density Function (PDF) of the centralized peak frequencies of the rebars; (d) center frequency normalization and depth correction; (e) data clustering; (f) GPR condition mapping.

As illustrated in Fig. 17, the analysis was applied to the reflection of each rebar to capture the center frequency values and rebar information (X-coordinates, Y-coordinates, and TWTT). The frequency values were normalized and depth-corrected based on Eq. (13) and Eq. (14), as depicted in Fig. 17 (d). The applied normalization and depth correction process bring the data to a uniform scale regardless of changes in cover depth values. Accordingly, to map the normalized and depth-corrected center frequency values, two threshold values were determined, as illustrated in Fig. 17 (e & f). Thus, the GPR condition mapping thresholds T1 and T2 were optimized to achieve the best agreement with the ground truth condition.

#### 5.2.2 Ground truth condition mapping

Ground truth conditions are essential to optimize and validate the GPR condition maps derived from the frequency-based method. Thus, ground truth conditions about corrosion and corrosion-induced delamination were collected. Accordingly, the hammer tapping test was used to evaluate delamination before rebars were exposed and visually inspected to evaluate corrosion, as shown in Fig. 6.

**Fig. 18:**
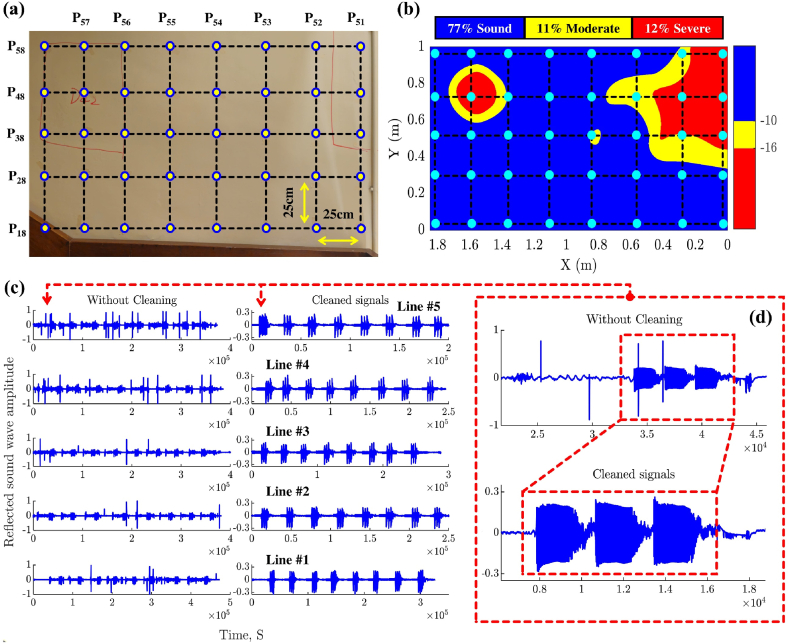
Experimental setup for the hammer-tapping test on the GPR-scanned concrete wall: (a) grid configuration of tested points, (b) projection of hammer-tapping points onto the GPR condition map, and (c) and (d) sound waves before and after noise removal and signal cleaning.

For precise referencing during ground truth condition mapping, a grid configuration with a spacing of 25 cm was employed for the hammer-tapping test and projected onto the GPR condition map, as shown in Fig. 18. The reflected sound waves emanating from every row within the grid were systematically recorded with the DXMIC PRO microphone in a single audio track, with three consecutive hammer taps at each point, as depicted in Fig. 18 (c & d). The three hammer taps were implemented to ensure redundancy and facilitate accurate differentiation of the tap signals from any extraneous noise caused by ambient sounds or the placement of the microphone probe at each test point. Accordingly, the noise in the audio track was effectively removed/filtered, as illustrated in Fig. 18 (d).

During sound recording, the noise was mainly generated by moving the probe from one testing point to another was easily identifiable and distinct from the signal of interest. To effectively filter out the effect of the noise, we employed a combination of segment analysis and visual inspection of sound waves. Accordingly, the audio track was divided into smaller, manageable segments, allowing for a detailed examination of each portion of the recording. By closely inspecting these segments, the noise peaks caused by the movement of the microphone probe (see Fig. 18) were identified, which typically appeared as sharp, transient spikes distinct from the consistent patterns of the hammer-tap signals. Once identified, these noise peaks were manually removed to ensure the integrity of the remaining signal. Additionally, the identified noise peaks were cross-referenced with the known positions of the microphone probe movements to confirm that the peaks were indeed due to probe movements and not part of the actual hammer-tap signals. By maintaining a log of the manual adjustments, consistency and reproducibility in the filtering process was ensured. This approach facilitated effective removal of noise from the audio track, resulting in a cleaner and more accurate representation of the reflected sound waves, thereby providing a reliable basis for further analysis and interpretation of the hammer-tapping test results.

**Fig. 19:**
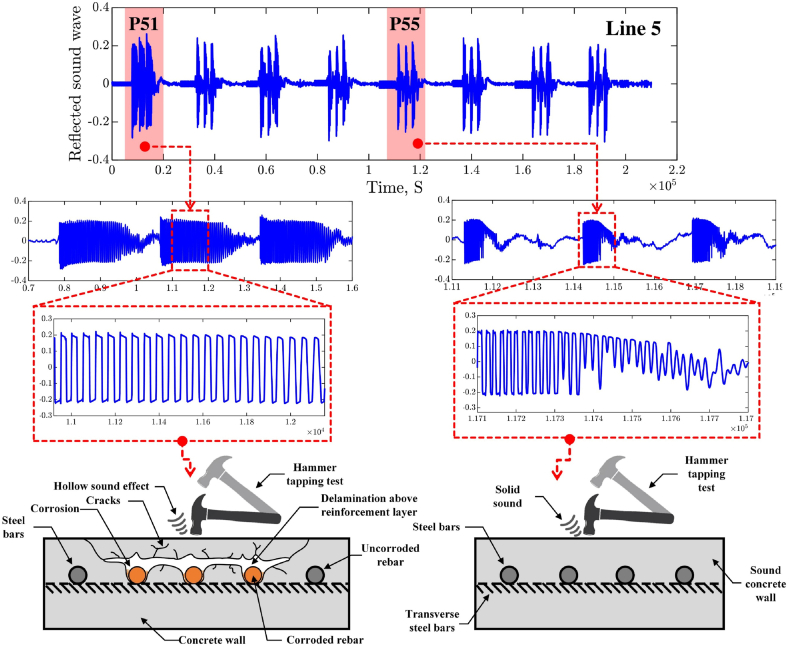
Comprehensive analysis and assessment of hammer tapping test for delaminated and sound concrete wall zones.

Subsequently, observing patterns across the three taps revealed that the acoustic behavior of concrete structures with air voids differs significantly from that of solid concrete walls, resulting in distinct sound reflection patterns. This phenomenon, rooted in the variations of acoustic impedance and density, has long been acknowledged as a valuable tool by experts in practical inspections for concrete wall assessment. Fig. 19 presents a detailed analysis and assessment of the hammer-tapping test to verify the accuracy of the generated GPR maps. This detailed examination includes a comparative analysis of the reflected sound waves obtained from sound and delaminated points.

When sound waves traveling through concrete encounter a medium with different acoustic impedance, part of the wave reflects back. The strength of this reflection depends on the contrast in impedance between the two media. A greater difference, such as between concrete and an air void, results in a stronger reflection. The points P_51_ and P_55_, initially depicted in Fig. 18, were presented as an example of the performed analysis. In Fig. 19, the acoustic profiles of the reflected sound waves originating from point P_51_ and point P_55_ offer distinctive characteristics that provide insights into the condition of the concrete walls under examination. For instance, at point P51, the strong reflection of sound waves indicates the presence of air voids within the structure. This strong reflection suggests that the sound waves are moving into a medium with relatively lower impediments, likely due to the presence of voids or gaps within the concrete material. Conversely, the acoustic response at point P_55_, located within a sound region and corresponding to a non-corroded segment, exhibits a damped reflection. The damped non-hollow sound observed at this point quickly decays, implying a more solid and homogeneous material composition without significant air voids or delamination. The rapid decay of sound waves indicates that the material at this location is more compact and cohesive, leading to the dissipation of the energy transmitted through the structure.

**Fig. 20:**
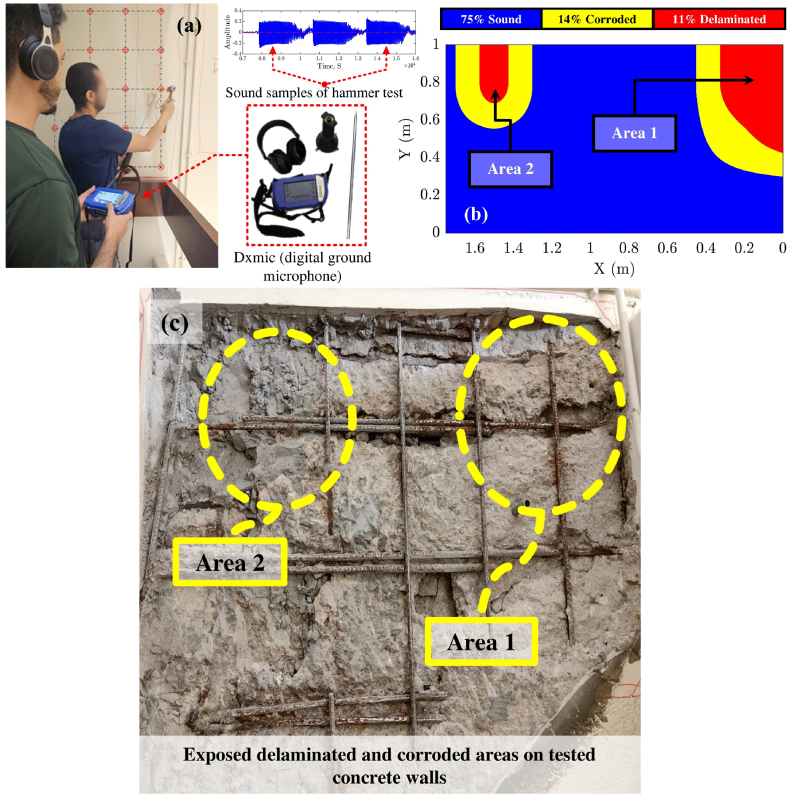
Ground truth map derived from the hammer tapping test and exposed rebars. (a) Test setup; (b) Generated ground truth map; (c) Exposed delaminated and corroded regions on the defected concrete wall.

After analyzing the data obtained from the hammer-tapping and uncovering the reinforcement rebars, a ground truth map depicting the locations of corrosion and delamination was produced using a grid of local coordinates, as shown in Fig. 20(b). In Fig. 20(c), images of the tested concrete wall are presented, highlighting select exposed delaminated and corroded zones (Area 1 and Area 2) within the concrete wall. The ground truth condition mapping detailed in this section was systematically used to optimize the GPR mapping threshold and validate the generated GPR condition maps.

#### 5.2.3 GPR thresholds optimization

**Fig. 21:**
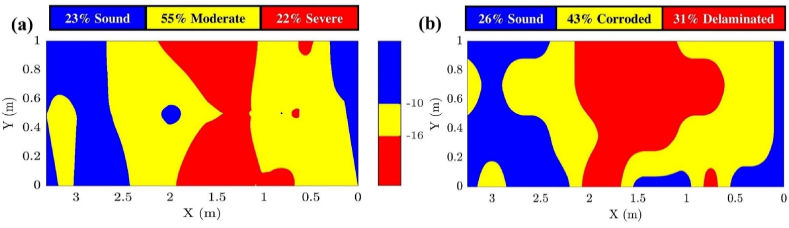
Condition mapping of the tested concrete wall: (a) GPR condition mapping; and (b) Ground truth map created from the hammer tapping test.

The GPR condition map from section 5.2.1 was cross-referenced with the ground truth condition map from section 5.2.2, as depicted in Fig. 21. Threshold optimization was then applied separately for T1 and T2. This process ensured the best alignment between the GPR condition map and the ground truth delamination and corrosion map, which were derived from the sounding test and reinforcement exposure. T1 distinguishes areas with active corrosion, while T2 identifies areas with severe corrosivity where corrosion-induced delamination might exist.

**Fig. 22:**
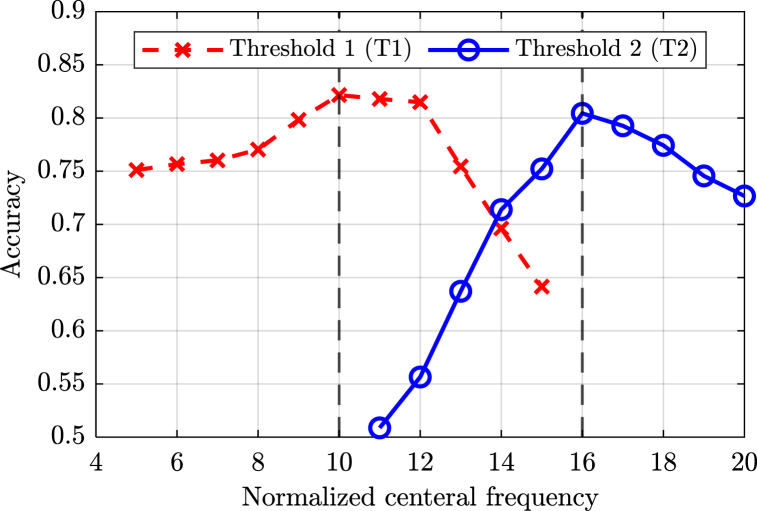
Threshold 1 and threshold 2 optimization curves.

During the optimization process, T1 was varied within the range of 8–15. For each threshold value, a GPR corrosiveness map was generated and cross-referenced with the ground truth corrosion map. Accordingly, Each pixel in the map was classified as follows: true positive (TP) if the pixel indicated corrosion in both maps, false positive (FP) if the pixel indicated active corrosion in the GPR map but not in the ground truth map, true negative (TN) if the pixel indicated soundness in both maps, and false negative (FN) if the pixel indicated soundness in the GPR map but corrosion in the ground truth map. The number of pixels in each classification was divided by the total number of pixels to obtain normalized percentages, as illustrated in Table 1. Accordingly, the accuracy was calculated for each T1 value using Equation (2). The optimal accuracy was achieved when T1 equaled 10, as illustrated in Fig. 22.

**Table 1:** Optimization metrics of the thresholds (T1 and T2) in the condition mapping.

Values of thresholds	TP %	FP %	TN %	FN %	Accuracy %
**T1: Thresholds 1**					
5	69.08	23.67	6.03	1.23	75.11
6	68.96	22.98	6.71	1.35	75.67
7	68.60	22.27	7.43	1.71	76.02
8	68.04	20.70	8.99	2.26	77.04
9	67.48	17.34	12.35	2.83	79.83
10	64.81	12.34	17.35	5.50	82.16
11	61.74	9.64	20.06	8.57	81.79
12	66.53	14.73	14.96	3.77	81.49
13	53.58	7.83	21.87	16.73	75.45
14	44.44	4.51	25.18	25.86	69.62
15	36.97	2.52	27.18	33.33	64.15
**T2: Thresholds 2**					
11	28.41	48.74	22.46	0.39	50.87
12	27.91	43.46	27.74	0.89	55.65
13	26.96	34.45	36.75	1.84	63.71
14	24.57	24.38	46.82	4.23	71.39
15	21.76	17.73	53.47	7.05	75.22
16	15.84	6.60	64.60	12.96	80.44
17	19.03	10.96	60.24	9.77	79.27
18	10.31	4.07	67.13	18.49	77.43
19	6.58	3.22	67.98	22.22	74.56
20	3.96	2.49	68.71	24.84	72.67

On the other hand, T2 was changed in the range between 10 and 17. For each threshold value, a GPR of severely corrosive areas that may exhibit corrosion-induced delamination was generated and compared with the ground truth delamination map. Each pixel in the map was classified into TP, FP, TN, and FN. The number of pixels in each classification was also divided by the total number of pixels to obtain normalized percentages, as illustrated in Table 1. The optimal accuracy was achieved when T2 equaled 16 (see Fig. 22).

According to the optimization process, the optimal GPR condition mapping thresholds were at T1 equals 10 and T2 equals 16. These two thresholds will be used to cluster and map the normalized and depth-corrected center frequency values, providing a representation of the active corrosion and corrosion-induced delamination conditions in the inspected reinforced concrete walls.

### 5.3 Model validation

In this section, the condition mapping of three reinforced concrete walls, previously identified as potentially exhibiting delamination and corrosion, was systematically generated using the method outlined in Section 4. The GPR condition maps were cross-referenced with the ground truth data to validate the accuracy of the proposed method in detecting both the intensity and extent of active corrosion (corrosivity) and severe corrosivity that may be linked to corrosion-induced delamination. To further assess the effectiveness of the proposed approach, the results from the frequency-based method are compared with those from the traditional amplitude-based method. The amplitude-based corrosivity assessment follows the methodology outlined by Faris et al. [65], which applies pairwise normalization and depth correction to rebar amplitudes and generates corrosivity mapping based on the statistical distribution of the normalized amplitudes.

**Fig. 23:**
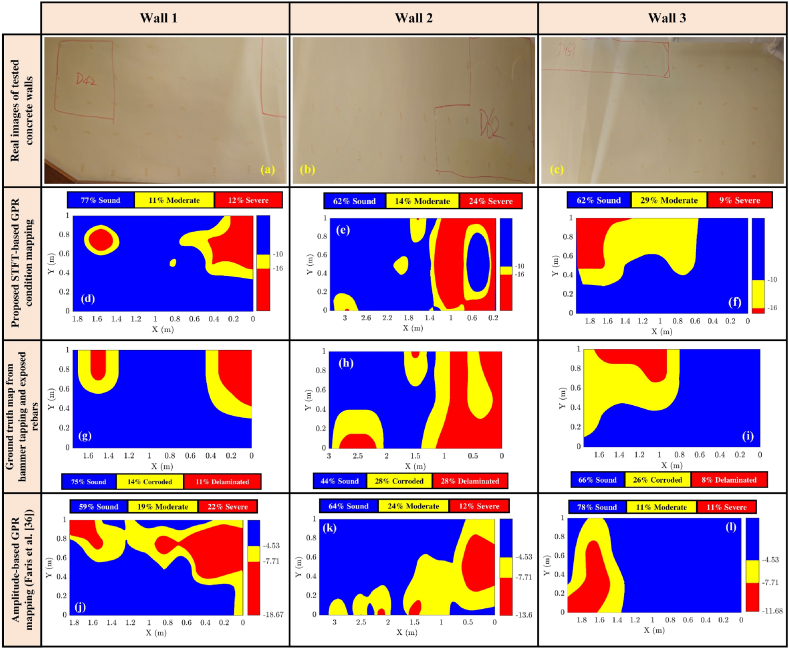
Comparison of GPR condition mapping approaches: (a), (b), and (c) real images of tested concrete walls with potential delamination/corrosion; (d), (e), and (f) GPR condition maps generated using the proposed STFT approach; (g), (h), and (i) ground truth maps based on hammer-tapping tests and exposed rebars; and (j), (k), and (l) GPR condition maps from amplitude analysis as per Faris et al. [65].

Fig. 23 illustrates the performance of the developed method for the three tested concrete walls and compares it to amplitude-based GPR condition mapping approaches. Fig. 23 (a, b, & c) displays real images of the walls, which are suspected of exhibiting delamination and corrosion. Fig. 23 (d, e, & f) shows the GPR condition maps generated using the proposed STFT approach, while Fig. 20 (g, h, & i) illustrates the ground truth maps derived from hammer-tapping tests and exposed rebars. Finally, Fig. 23 (j, k, & l) depicts the GPR condition maps generated using the amplitude analysis method based on Faris et al. [65]. This figure enables a direct comparison of the proposed methodology with traditional approaches, highlighting the differences in detected deterioration patterns and providing insights into the accuracy and reliability of each method.

As illustrated in Fig. 23 (d, e, & f), the proposed method showed strong performance in detecting deterioration in reinforced concrete walls. As detailed in Table 2, the average accuracy for active corrosion (corrosivity) detection was 0.80, while the accuracy for detecting severe corrosivity, specifically corrosion-induced delamination, was 0.84. The model demonstrated superior performance in detecting severe corrosivity with corrosion-induced delamination compared to active corrosion. This phenomenon can be attributed to the presence of excess moisture and chlorides trapped within the delaminated areas, which cause significant attenuation of the high-frequency components of GPR waves, thereby improving the detection of delamination.

**Table 2:** GPR condition mapping performance.

Wall	Ground truth	GPR	TP	FN	FP	TN	Accuracy
Wall 1	Corrosion	Moderate	16.17	3.09	70.92	9.82	0.87
	Delamination	Severe	0.53	2.12	95.12	2.24	0.96
Wall 2	Corrosion	Moderate	36.14	1.82	44.07	17.97	0.80
	Delamination	Severe	13.18	10.66	68.01	8.15	0.81
Wall 3	Corrosion	Moderate	26.12	23.53	44.41	5.94	0.70
	Delamination	Severe	1.63	22.19	72.81	3.37	0.74

Fig. 23 (j, k, & l) presents the condition maps generated using the amplitude analysis approach outlined by Faris et al. [65], applied to Wall 1, Wall 2, and Wall 3. To facilitate the comparison, the last two thresholds in Faris's method were combined into a single threshold. Similarly, the condition maps used blue, yellow, and red to represent areas in sound, moderate, and severe conditions, respectively. As shown in Fig. 23, the locations of the deteriorated areas identified by the amplitude-based method are less aligned with the ground truth compared to the frequency-based maps generated by the proposed method. For example, in Wall 3, the predicted corroded areas are incorrectly concentrated in the bottom-right corner, whereas the actual deterioration is located in the top-right corner.

The reduced performance of the amplitude-based method can be attributed to the differences between concrete walls and concrete bridge decks, as the Faris et al. [36] method was originally designed for bridge decks. Specifically, the smaller surface area of concrete walls makes pairwise normalization and depth correction more sensitive to local variations in the test area. Additionally, the thinner cover depth of concrete walls increases the influence of ground reflections on the amplitude of rebar reflections, leading to overlapping signals that make the amplitude values less representative of the actual rebar reflections.

The findings highlight the improved accuracy of the proposed method in assessing corrosivity, offering a more reliable evaluation of the severity and extent of corrossivity. This enhancement suggests that the developed approach holds potential for practical applications in condition monitoring. However, this study was limited to a 1.6 GHz GPR antenna and data from reinforced concrete walls. Furthermore, the method does not separately evaluate the distinct effects of chlorides, moisture, and corrosive byproducts. Therefore, further research is needed to assess the generalizability and performance of the method across a broader range of concrete structures and GPR frequencies, especially in diverse environmental conditions and structural configurations.

## 6 Conclusion

GPR is a prominent NDT method for assessing the corrosivity of concrete structures. Traditional GPR interpretation in the context of corrosivity evaluation relies heavily on the absolute value of rebar reflection energy, which is a result of complex influencing factors. This study introduced a novel method for GPR interpretation using time and time-frequency domain analysis. Key innovations included simulated ground reflection filtering to facilitate analysis in shallow cover depths, along with automated frequency-domain analysis leveraging STFT and DL algorithms. Accordingly, the center frequency of rebar reflections was normalized and depth-corrected to standardize the analysis and GPR condition mapping. The thresholds for GPR condition mapping were optimized to achieve the best alignment with ground truth conditions.

The method demonstrated high performance in automated rebar recognition and subsequent GPR condition mapping. The DL rebar recognition demonstrated a strong performance with a precision and recall of 0.94 and 0.93, respectively. In addition, GPR condition mapping outperformed the traditional amplitude-based method with an average accuracy of 0.80 for active corrosion detection and 0.84 for detecting severe corrosivity with corrosion-induced delamination. This can be attributed to that the traditional amplitude-based methods have limitations in evaluating structures with small areas and small cover depths, such as concrete walls. The enhanced performance of the developed method is also attributable to the adopted pre-processing and filtering techniques and the comprehensivity of the time-frequency analysis.

The findings confirm the effectiveness of the frequency-based method in improving the reliability of GPR-based corrosivity assessment, which in turn supports more informed maintenance and intervention decisions for concrete structures. The method proved its efficiency in assessing concrete structures with shallow cover depth. However, this study was limited to a constant frequency of 1.6 GHz and data collected from concrete walls. Furthermore, the method does not separately evaluate the distinct effects of chlorides, moisture, and corrosion byproducts. Thus, if corrosion is not active, the method may face challenges in detecting corrosion byproducts. To address these limitations, future research should monitor variations in all frequency components instead of focusing only on the center frequency shifts. Thus, DL and ML methods could provide deeper insights into the distinct effects of chlorides, moisture, and corrosive byproducts. Additionally, extend the methodology to broader case studies, such as concrete bridge decks, and assess its generalization across different GPR antennas to evaluate the impact of antenna specifications on the results. Moreover, incorporating complementary NDE techniques, such as impact echo, could improve the detection of smaller or deeper delamination that may not strongly affect the GPR signal.

## CRediT authorship contribution statement

**Nour Faris:** Formal analysis, Investigation, Methodology, Software, Validation, Writing – original draft. **Ahmed K. Khalil:** Formal analysis, Investigation, Methodology, Software, Validation, Writing – original draft. **Mohamed A.A. Abdelkareem:** Formal analysis, Investigation, Methodology, Software, Validation, Writing – original draft. **Sherif Abdelkhalek:** Visualization, Writing – review & editing. **Ali Fares:** Visualization, Writing – review & editing. **Tarek Zayed:** Funding acquisition, Project administration, Supervision, Writing – review & editing. **Ghasan Alfalah:** Funding acquisition, Writing – review & editing.

## Declaration of competing interest

The authors declared no potential conflicts of interest with respect to the research, authorship, and/or publication of this article. This manuscript has not been published and is not under consideration for publication elsewhere. All authors have read the manuscript and have approved this submission. The authors report no conflicts of interest. The authors also confirm that any necessary permissions have been obtained.
